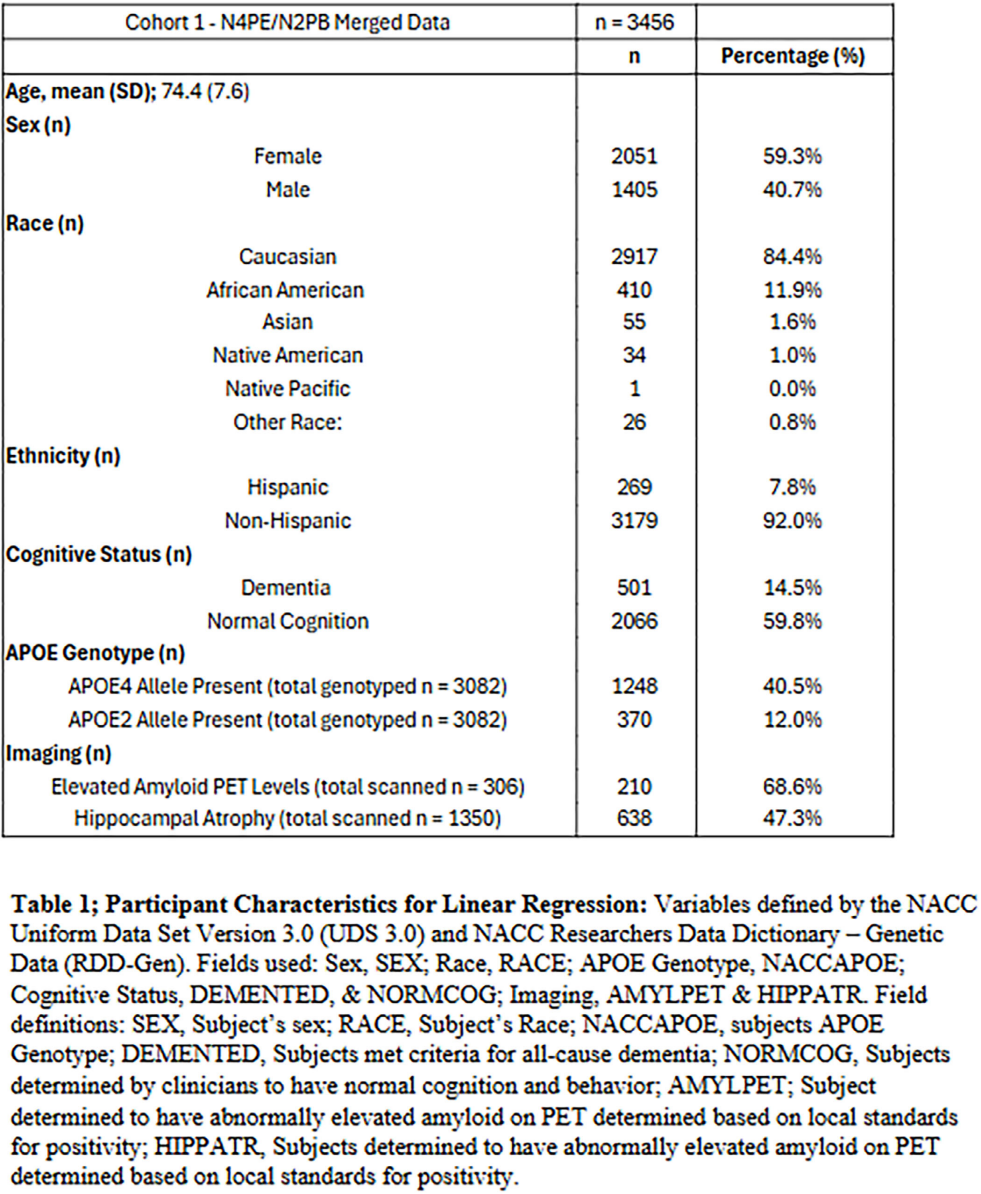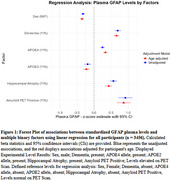# Cross‐Sectional Analysis of Plasma GFAP Across Alzheimer's Disease Stages

**DOI:** 10.1002/alz70856_106901

**Published:** 2026-01-08

**Authors:** John R. Hoffman, Kristen A. Russ, Alex J. Schwefel, Tatiana M. Foroud, Naazneen Khan, Yi Zhao, Jeffrey L. Dage

**Affiliations:** ^1^ Medical Neuroscience Department, Indiana University School of Medicine, Indianapolis, IN, USA; ^2^ Department of Medical and Molecular Genetics, Indiana University School of Medicine, Indianapolis, IN, USA; ^3^ National Centralized Repository for Alzheimer's Disease and Related Dementias (NCRAD), Indianapolis, IN, USA; ^4^ Indiana University School of Medicine/NCRAD, Indianapolis, IN, USA; ^5^ Department of Neurology, Indiana University School of Medicine, Indianapolis, IN, USA; ^6^ Department of Biostatistics & Health Data Sciences, Indiana University School of Medicine, Indianapolis, IN, USA; ^7^ Department of Neurology, Indiana University School of Medicine, Indianopolis, IN, USA

## Abstract

**Background:**

Alzheimer's Disease (AD) is the leading cause of dementia worldwide, with Late‐Onset Alzheimer's Disease (LOAD) comprising over 90% of cases. Multiple biomarkers are being investigated for their association with pathologies associated with AD including glial acidic fibrillary protein (GFAP). GFAP levels have been observed to increase in plasma and cerebrospinal fluid of AD patients, particularly in response to amyloid‐β, before behavioral symptoms appear. This indicates GFAP's utility as an early‐AD biomarker. However, GFAP levels vary significantly between subjects due to various factors, such as demographics or medical comorbidities. To use plasma GFAP levels as an accurate biomarker in AD studies, it is essential to fully understand and identify all potential reasons for variation in its levels.

**Method:**

The uniform data set (UDS), which contains longitudinal subject data from AD Centers (ADC) was obtained from the National Alzheimer's Coordinating Center (NACC). Total GFAP levels from 23 ADC's were measured by the National Centralized Repository for Alzheimer's Disease and Related Dementias (NCRAD) using Quanterix Simoa‐HDx (N4PE and N2PB). Results were log10‐transformed and z‐scored for analysis. A total of 3,456 unique subjects at a single visit, aged 60+, were included in this cross‐sectional analysis. T‐tests, one‐way ANOVAs, Tukey's‐ HSD Post‐Hoc and linear regression were used to examine the association of GFAP levels with diagnosis, pathology, demographics and comorbid conditions.

**Result:**

Analysis showed that GFAP levels varied significantly (*p*‐value < 0.05) with age in addition to sex, APOE genotype, elevated amyloid PET, hippocampal atrophy, and cognitive status. The non‐demographic associations were not significantly modified when adjusting for the subjects' age despite a significant association of GFAP levels with age overall.

**Conclusion:**

Plasma GFAP levels differed significantly between males and females, emphasizing the need to account for sex differences in biomarker research. Plasma GFAP levels show strong associations with risk alleles, elevated amyloid PET levels, hippocampal atrophy, and cognitive status, supporting its potential as a biomarker for AD diagnosis. Future efforts will focus on exploring the impact of changes in GFAP on changes in atrophy, cognitive decline, and how these relationships are modified by comorbid conditions.